# Antimicrobial Resistance and Molecular Epidemiological Characteristics of Methicillin-Resistant and Susceptible Staphylococcal Isolates from Oral Cavity of Dental Patients and Staff in Northern Japan

**DOI:** 10.3390/antibiotics10111316

**Published:** 2021-10-29

**Authors:** Mina Hirose, Meiji Soe Aung, Atsushi Fukuda, Shoko Yahata, Yusuke Fujita, Masato Saitoh, Yukito Hirose, Noriko Urushibara, Nobumichi Kobayashi

**Affiliations:** 1 Division of Pediatric Dentistry, Department of Oral Growth and Development, School of Dentistry, Health Sciences University of Hokkaido, Ishikari-Tobetsu 061-0293, Japan; minaniwa@hoku-iryo-u.ac.jp (M.H.); atsushi@hoku-iryo-u.ac.jp (A.F.); 8.yaha.24@gmail.com (S.Y.); fujita-y@hoku-iryo-u.ac.jp (Y.F.); msaitoh@hoku-iryo-u.ac.jp (M.S.); 2 Department of Hygiene, Sapporo Medical University School of Medicine, Sapporo 060-8556, Japan; noriko-u@sapmed.ac.jp (N.U.); nkobayas@sapmed.ac.jp (N.K.); 3 Division of Fixed Prosthodontics and Oral Implantology, Department of Oral Rehabilitation, School of Dentistry, Health Sciences University of Hokkaido, Ishikari-Tobetsu 061-0293, Japan; yukito@hoku-iryo-u.ac.jp

**Keywords:** MRSA, *Staphylococcus argenteus*, coagulase-negative *Staphylococcus*, oral cavity, hand, SCC*mec*, PVL, enterotoxin, ACME

## Abstract

The acquisition of drug resistance and virulence by staphylococcal species colonizing humans is a growing public health concern. The present study was conducted to investigate the prevalence, antimicrobial resistance and genetic characteristics of *Staphylococcus* isolates from the oral cavity and skin (hand) of systemically healthy subjects with dental disease and dental staff in northern Japan. Among a total of 133 subjects (91 patients and 42 staff), 87 coagulase-positive *Staphylococcus* (83 *S. aureus*/4 *S. argenteus*) and 162 coagulase-negative *Staphylococcus* (CoNS) isolates were recovered from 59 (44.4%) and 95 (71.4%) subjects, respectively. Three oral isolates were methicillin-resistant *S. aureus* (MRSA) (3.6%, 3/83) that were genotyped as ST8-SCC*mec*-IVl, ST4775(CC1)-SCC*mec*-IVa and ST6562(CC8)-SCC*mec*-IVa. Remarkably, the ST6562 isolate harbored PVL genes on ΦSa2usa and type I ACME (arginine catabolic mobile element). Four methicillin-susceptible isolates were identified as *S. argenteus* belonging to ST1223 and ST2250, which harbored enterotoxin genes *egc-2* and *sey*, respectively. Among the fourteen CoNS species identified, methicillin-resistant (MR) isolates were detected in five species (11 isolates, 13.3% of CoNS), with *S. saprophyticus* and *S. haemolyticus* being the most common. ACME was prevalent in only *S. epidermidis* and *S. capitis*. These findings indicated the potential distribution of USA300 clone-like MRSA, toxigenic *S. argenteus* and MR-CoNS in the oral cavity of dental patients.

## 1. Introduction

*Staphylococcus* is well-known commensal bacteria in humans and most mammals and forms the normal flora of the skin and mucous membrane. This genus has been divided into coagulase-positive and -negative groups that include more than forty species [1]. Human-associated coagulase-positive *Staphylococcus* (CoPS) represents more pathogenic groups of *Staphylococcus*, comprising at least three species, among which *S. aureus* is the most common cause of a wide variety of diseases including skin and soft tissue infections, pneumonia, bacteremia, septic shock, food poisoning and toxic shock syndrome [2]. *S. argenteus* and *S. schweitzeri*, which were previously included in *S. aureus*, were reclassified as new species of CoPS in 2015 [3]. In particular, *S. argenteus* has been recognized as an emerging pathogen distributed to humans and animals that causes various diseases, such as *S. aureus*, and reported worldwide [4]. *S. aureus* and *S. argenteus* produce diverse virulence factors, such as exotoxins (enterotoxins, exfoliative toxins, etc.), exoenzymes and adhesins, a part of which are associated with various symptoms due to infections of these organisms [5,6]. While coagulase-negative *Staphylococcus* (CoNS) is regarded as a less virulent group, some CoNS species are common opportunistic pathogens that pose a significant health burden [2]. Nosocomial infections with some CoNS species become difficult to treat, due to the ability of biofilm to form on indwelling medical devices [7].

During the past two decades, the global spread of methicillin-resistant *S. aureus* (MRSA) in the community has also been noted as a cause of disease in immunocompetent individuals, while it had traditionally been confined to hospital settings [8]. Similarly, methicillin-resistant CoNS (MR-CoNS) has also been increasingly recognized as a nosocomial pathogen in many species [2]. In its chromosome, methicillin-resistant *Staphylococcus* carries the staphylococcal cassette chromosome *mec* (SCC*mec*), which contains *mecA* encoding penicillin-binding protein 2a with low affinity to beta-lactams. The SCC*mec* of MRSA has been classified into 14 genotypes (I-XIV) [9,10], among which types I, II, III, IV and V are commonly reported for healthcare-associated (HA)-MRSA or community-associated (CA)-MRSA. The acquisition of virulence factors in CA-MRSA, which may imply an increased virulence, is a potential concern for public health. For example, a CA-MRSA clone USA300 that has been predominant in the US, characteristically produces Panton Valentine leukocidine (PVL) associated with severe symptoms and has an arginine catabolic mobile element (ACME) in its genome that is implicated with an increased adaptability to human skin [8,11].

The colonization of *S. aureus*/MRSA on anterior nares is associated with the pathogenesis of their infections [12], increasing the risk of bloodstream infections and other infections resulting in an elevated medical burden [13,14]. Similarly, the carriage of CoNS in skin is implicated in surgical site infections [15,16], and the spread of MR- and multidrug resistant CoNS among healthy individuals has been described [17]. Thus, the colonization of *Staphylococcus* has been characterized mainly for bacterial strains residing in the nasal cavity and the skin, which are considered the primary ecological niche of this genus. Although 12–30% of individuals carry *S. aureus* in their nasal cavity persistently, 30% (range 16–70%) are intermittent carriers [18]. In contrast, colonization in the oral cavity is considered more persistent [19,20], and the reported carriage rate of *S. aureus* in the oral cavity is almost similar to or higher than those in nare in some studies [21,22]. Furthermore, colonizing *S. aureus* was only detected in the oral cavity in a substantial part (approximately 25%) of carriers [23,24]. Accordingly, the oral cavity/oro-pharynx has been noted and described as a more significant reservoir of staphylococci than anterior nares, for lower respiratory infections, cross-infection and dissemination to other body sites [20,25,26,27,28].

In our previous study, the prevalence of MRSA and MR-CoNS in the oral cavity of healthy children and their genetic traits were analyzed in Hokkaido, the northern main island of Japan [29]. As a result, 6.3 and 50% of colonizing *S. aureus* and *S. epidermidis* were identified as being MR, respectively, with MRSA belonging to five STs (ST1, ST5, ST8, ST89 and ST120). The present study was conducted to investigate the drug resistance and genetic characteristics of oral staphylococcal isolates from subjects of all age groups (systematically healthy subjects with dental disease and dental staff) and compared with isolates from the skin of the subjects’ hand, to understand the spread of bacterial strain within a subject. The results of this study provided different features in the prevalence of oral MR and methicillin-susceptible (MS) staphylococcus, with the first identification of USA300 clone-like MRSA and MS *S. argenteus* in an oral cavity.

## 2. Results

### 2.1. Prevalence of Staphylococcal Isolates from Study Subjects

During a 15-month period starting in December 2019, a total of 133 subjects participated in this study. They consisted of 91 systemically healthy dental patients (74 and 17 subjects with mild and severe dental disease, respectively) and 42 dental staff (35 dentists and 7 dental hygienists). The age range of participants was 0–93 years (median age: 46.5 years), and sex ratio was 0.99 (Table S1). Seventy percent of the patients with mild disease were children (0–18 years), while most of those with severe disease were adults. For a bacterial culture, a saliva and a skin swab on the hand (fingers and palm) were taken from all the subjects, and additionally, a swab of the oral disease site was collected from the patients with severe disease. During the study period, there were few or no participants from March to June 2020 because of the decrease in dental patients caused by the COVID-19 pandemic. However, in other periods, the monthly number of study subjects was almost constant.

Among the 133 subjects, two CoPS species, *S. aureus* and *S. argenteus*, were isolated from 63 (47.4%) and 3 (2.3%) subjects, respectively, while CoNS was isolated from 95 subjects (71.4%). The CoPS-positive rates were higher in the dental patient groups (overall rate: 47.3%, 43/91) than in the staff (38.1%) (statistically not significant; *p* = 0.32) (Table 1). From any sampling sites of all the subjects, a total of 83 *S. aureus*, 4 *S. argenteus* and 162 CoNS isolates were recovered. CoPS was more frequently isolated from the oral cavity (58 isolates) than the hand (29 isolates), though 87% of CoNS isolates (141/162) were derived from a skin swab of the hand. Three MRSA and 11 MR-CoNS isolates were identified, showing detection rates of 3.6 and 8.3% in *S. aureus* and CoNS, respectively. *S. argenteus* isolates were all *mecA*-negative. MRSA was only isolated from the oral cavity (staff and dental patients with mild disease), while most of MR-CoNS isolates were recovered from the hand of patients.

### 2.2. Genotypes, Antimicrobial Resistance, Virulence Factors and Resistance Genes in CoPS

Eighty-three *S. aureus* isolates were classified into 11 coagulase genotypes (*coa*-types) and 20 STs, with *coa*Xa/ST15 being the most common (11 isolates), followed by *coa*-IIIa/ST8 and *coa*-VIc/ST97 (10 isolates each), and *coa*-IVa/ST30 and *coa*-Vb/ST188 (8 isolates each) (Table 2). Three MRSA isolates were genotyped as ST8-SCC*mec* IVl, ST4775-SCC*mec* IVa and ST6562(CC8)-SCC*mec* IVa. ST4775 and ST6562 are single locus variants of ST1 (allelic profile: 1-712-1-1-1-1-1) and ST8 (allelic profile: 3-3-1-1-4-739-3), belonging to clonal complex (CC) 1 and CC8, respectively. The ST6562 isolate belonged to *spa* type t1188 having a repeat profile (11-19-12-21-34-24-34-22-25) similar to that of t008 (11-19-12-21-17-34-24-34-22-25).

Among the 18 antimicrobials examined for *S. aureus*, the highest resistance rate was found against AMP (45.8%), followed by ERY (24.1%), and CLI (20.5%) (Table S2). All the ST30 isolates showed resistance to these three antimicrobials, harboring *blaZ* and *erm(A)* (Table 2, Table S2). A high resistance rate to AMP was observed for ST15, ST20 and ST121, among which ST15 and ST121 included GEN-resistant isolates harboring *aac(6′)-Ie-aph(2”)-Ia*. Three MRSA isolates showed multidrug resistance having any of the aminoglycoside modifying enzyme genes and macrolide resistance genes. The ST6562 isolate had *blaZ*, *aph(3′)-IIIa* and *msr(A)*. All the isolates are sensitive to LZD with MIC ranging between 0 and 2 μg/mL and none of the isolates harbored *optrA*, *fexA* and *cfr* genes.

The prevalence of virulence factors was analyzed in 38 *S. aureus* isolates with different genotypes and four *S. argenteus* isolates (Table S3). ST6562 (CC8) MRSA harbored PVL genes (*lukS-PV-lukF-PV*) located on a phage ΦSa2usa and had also type I ACME that included *speG* encoding spermine/spermidine N-acetyltransferase as typically seen in the USA300 clone [30], while ST8/SCC*mec*-IVl MRSA had *tst-1*, *sec*, *sep* and also *spj* encoding a cell wall-anchored protein unique to SCC*mec* IVl [31]. An enterotoxin gene cluster (*egc*: *egc-1*, *seg-sei-sem-sen-seo*; *egc-2*, *seg-sei-sem-sen-seo-seu*) was identified in various genotypes of MSSA, including ST20, ST30 and ST121, among which ST30 isolates also harbored *tst-1*. Enterotoxin genes *sea* and *seb* were present in ST8 and ST12 MSSA isolates, respectively. Exfoliative toxin genes *eta* or *etd* were detected in MSSA isolates with ST26, ST121, ST291 and ST1607. A variant gene of the elastin-binding protein (*ebpS-v*) [32] was only identified in ST121 isolates.

Four *S. argenteus* isolates were genotyped as *coa*-XV/ST1223 (two isolates) and *coa*-XId/ST2250 (two isolates), which carried *egc-2* and *sey*, respectively. These showed susceptibility to all of the 18 antimicrobials examined.

*S. aureus* or *S. argenteus* were recovered from both the oral cavity (saliva, disease site) and the hand of 19 subjects (Table 3). Among them, isolates from the two sites of the 13 subjects showed identical genotypes and other genetic traits, and resistance profiles. The presence of ST2250 *S. argenteus* in saliva and on the hand was confirmed in a patient (12 years, female) with mild dental disease. In a single subject (B20-H05) with mild dental disease, though the isolates from saliva and the hand belonged to the same genotype (*coa*-X/ST15), they showed different profiles of resistance and their responsible genes.

### 2.3. CoNS Species, Prevalence of MR-CoNS and Antimicrobial Resistance

A total of 162 CoNS isolates were differentiated into 14 species, with *S. warneri* and *S. capitis* being dominant (35.8 and 32.1%, respectively) and prevalent mainly on the hand (Table 4). These two species and four other common species, i.e., *S. saprophyticus*, *S. epidermidis*, *S. caprae* and *S. haemolyticus*, accounted for 90% of all the CoNS isolates. MR-CoNS was detected in 13.3% (11 isolates; two from the oral cavity, nine from the hand) of all the isolates in the five species, with *S. saprophyticus* and *S. haemolyticus* being the most common. The SCC*mec* of MR-CoNS was mostly non-typeable (Table S4). ACME was only detected in *S. epidermidis* and *S. capitis*, with detection rates of 66.7 and 50%, respectively. The highest resistance rate was observed against FOF (46.9%), followed by ERY (21%) and AMP (16.7%) (Table S5). FOF resistance was common in *S. warneri*, *S. capitis*, *S. saprophyticus*, *S. haemolyticus* and *S. caprae*. Resistance to GEN was found mostly in *S. warneri*, and LVX resistance was only detected in *S. saprophyticus* and *S. haemolyticus*. In MR-CoNS isolates, the macrolide resistance genes (*erm(A)*, *erm(B)*, *erm(C)*, or *msrA*) were commonly detected, in addition to the most prevalent *blaZ* (Table S6).

## 3. Discussion

In the present study, we first revealed the prevalence and genotypes (*coa*-type, ST) of MRSA and MSSA from the oral cavity of dental patients, and also the spectrum of CoNS species with their resistance phenotype and determinants. The prevalence of *S. aureus* and MRSA in an oral cavity reported to date varies depending on the study design having different subjects [33]. In four studies on systemically healthy dental patients, the isolation rate of *S. aureus* ranged from 5.9 to 36.6%, while MRSA 0–8.6% [34,35,36,37]. For other study subjects, the rates of oral *S. aureus* were described as 38–40% in admitted patients [21,24], 35–48% in healthcare workers [24,38], 15–45% in healthy dental students [22,34] and 26–36% in healthy children [23,28,29]. A study in Ireland showed a lower rate of *S. aureus* isolation from patients than healthcare workers, while there was a slightly higher rate of MRSA in patients [24]. The oral carriage rates of MRSA in healthy subjects were 1.9% (dental students) [22] and 4.1% (healthcare workers) [38], with MRSA accounting for 9–21% of colonizing *S. aureus*. Among the *S. aureus* from oral specimens, approximately 10% of the isolates were identified as MRSA [26]. In our present study aimed at dental patients and staff, the overall isolation rate of *S. aureus* (44.4%) represented a high level compared with those reported for dental patients as well as healthy individuals as described above. However, MRSA was detected at a low rate among study subjects (2.3%, 3/133), which was similar to that in our previous study on healthy children (MRSA rate, 1.7%) [29]. Thus, it is suggested that the low prevalence of MRSA career (approx. 2%) has been persisting in our study site. In addition, though the isolation rates of *S. aureus* were slightly higher in the dental patients than the dental staff, this difference was not statistically significant, and MRSA was identified in both groups. Accordingly, it seems that *S. aureus* and MRSA may be distributed evenly to the oral cavity of the two groups of subjects.

Our present study revealed the clonal structure of *S. aureus* from the oral cavity and showed the broad genetic diversity of MSSA with common genotypes *coa*-IIIa (ST8), *coa*-IVa (ST30), *coa*-Vb (ST188), *coa*-VIc (ST97), *coa*-VIIa (ST12) and *coa*-Xa (ST15). Among them, ST8, ST30 and ST15 were considered to be persistently prevalent because they were also found in the oral cavity of healthy children in our previous study [29]. Furthermore, ST12, ST15, ST20, CC45, ST97 and CC398, which accounted for 51% (42/83) of all *S. aureus* isolates, have been described as common genotypes among livestock-associated MSSA/MRSA [39,40,41]. This finding suggests that in the present study population, an unexpectedly large part of the *S. aureus* isolates in the oral cavity were presumably transmitted from animals followed by colonization, due to their reduced pathogenicity to humans. Most of the MSSA isolates were generally susceptible to the antimicrobials tested and harbored less virulence factors, suggesting less pathogenic significance to humans. However, several clones, including ST8, ST12, ST30 and ST121, harbored *sea*, *seb*, *egc*, *tst-1*, *eta* or *etd*, which are related to pathogenicity in staphylococcal infections [5,6]. In addition, the concomitant distribution of the same *S. aureus* clones to the oral cavity and the hand was confirmed in approx. 70% of the subjects demonstrating *S. aureus* in both sites. These observations may indicate that the oral cavity has a potential role as a reservoir of *S. aureus* that mediates disease in humans, through transmission via oral droplet or the direct contact of body sites contaminated with this microorganism.

Among the clinical isolates of MRSA from healthcare and community settings in Hokkaido, Japan, *coa*-IIa (CC5) is predominant, and *coa*-IIIa (CC8) and *coa*-VIIa (CC1, CC59) are also commonly detected [42,43,44]. Unlike these dominant clones, three MRSA isolates in the present study (ST8-SCC*mec* IVl, ST4775-SCC*mec* IVa, ST6562-SCC*mec* IVa) had unique characteristics. ST4775, a single-locus variant of ST1, was reported previously in only an MRSA with an SCC*mec* IVa strain from a pet cat in Japan [45], suggesting its potential relation to the animal. The ST8-SCC*mec* IVl isolate, which was derived from a young dental patient (13y) in the present study, was PVL-negative and harbored *sec*, *sep*, *tst-1* and *spj*, which was characteristic of the CA-MRSA/J clone that emerged in Japan in 2003 as a cause of skin infections among children [31]. Despite the low prevalence, this clone has been identified among the CA- and HA-MRSA isolated from all ages in the Japanese population, occasionally leading to severe diseases [42,43,44,46,47]. Moreover, this clone was demonstrated in the oral cavity of healthy children in our previous study [29]. Therefore, it is conceivable that CA-MRSA/J has been potentially spread among the community in our study site. The colonizing nature as well as the pathogenic traits of this clone are suggested to be ascribable to the unique adhesin, a cell wall-anchored surface protein (CWASP/J) encoded by *spj* carried within SCCmec IVl [31,48]. A remarkable finding in our present study was the identification of ST6562 SCC*mec* IVa MRSA harboring PVL genes in Φsa2usa and ACME-I, which are typical traits of the USA300 clone [30]. ST6562 is a novel ST representing a single locus variant of ST8, and this isolate has a *spa* type closely related to that of the ST8 USA300 clone. Accordingly, the ST6562 MRSA is considered to be a genetic variant that originated from the USA300 clone. As a dominant CA-MRSA in the US, the USA300 clone has been evidently recognized since 2000, followed by global dissemination [49,50]. In Japan, since the first detection of this clone in 2007 [51], a low prevalence of this clone (0.2–3.1%) has been reported in community and healthcare settings [42,43,52,53]. Nevertheless, an increasing trend was also noted [53], and 5.1% of the isolates from blood samples were identified as this clone in our recent study [44]. Therefore, associated with the persistent spread of the USA300 clone among the Japanese population, ST6562 is suggested to have emerged as a local variant. Although the nasal colonization of the USA300 clone was described as a risk of infection route [54,55], the oral carriage of this clone has been scarcely reported, while only a report showed its colonization on the throat [56]. Our finding may indicate that the oral cavity should also be considered as a reservoir of such virulent MRSA clones.

It was notable that two genotypes of *S. argenteus* (ST1223, ST2250) were identified firstly in the oral cavity, in three subjects, with one carrying the same clone on their hand, while the nasal colonization of *S. argenteus* was demonstrated in tropical regions [57,58]. Except in highly endemic regions (Australia, Southeast Asia, Amazon), the prevalence of *S. argenteus* is very low [4]. In northern Japan, we reported that the frequency of *S. argenteus* corresponded to 0.6–0.7% of the total number of *S. aureus* clinical isolates [6,59]. In contrast, the incidence of colonizing *S. argenteus*, i.e., 5% of subjects (3/59) and 4.6% of isolated CoPS (4/87), in the present study appear to be remarkably high, suggesting that *S. argenteus* is prone to be carried asymptomatically by humans. As shown in the present study, the ST1223 and ST2250 strains specifically possess *egc* and *sey*, respectively [6,59], and a food poisoning outbreak due to ST1223 strain harboring *seb* was reported [60]. More research is necessary to evaluate *S. argenteus* colonization as a potential risk of disease in humans.

In our study, CoNS isolates were more commonly recovered from the hand than the oral cavity, and the frequencies by species were different from those observed for clinical isolates, with *S. epidemidis* being the most common and including more MR strains than other species [1,61]. In a German study, the *mecA*-positive rate of CoNS from the nasal cavity was 7% [17], which is comparable to that in our study (8.3%), although the composition of the CoNS species was considerably different. Though our CoNS isolates from the oral cavity/hand showed a susceptibility to most antimicrobials, a relatively high resistance rate (46.9%) was noted against FOF (fosfomycin), especially in *S. warneri* and *S. capitis*, in contrast to the susceptibility in all the *S. aureus* isolates. FOF resistance in staphylococcus is mediated by either a defective transporter with mutation in the chromosomal genes or the plasmid-associated FOF-inactivating enzyme, FosB [62]. Moreover, diverse variants have been known for the *fosB* gene depending on the staphylococcal species [63]. The identification of the *fosB* in the two CoNS species with FOF resistance is necessary to define its origin. Among CoNS, ACME has been most commonly identified in *S. epidermidis* clinical isolates [61,64]. However, in our study, *S. capitis* showed a prevalence of ACME (50%) comparable to *S. epidemidis* (67%), suggesting its increased colonizing ability. Accordingly, the genetic and phenotypic traits of *S. capitis* should be carefully monitored.

In conclusion, we revealed the prevalence, antimicrobial resistance and genetic characteristics of *Staphylococcus* from the oral cavity of dental patients and staff in northern Japan. PVL-positive USA300 clone-like MRSA, ST1223 and ST2250 *S. argenteus* were first identified as orally colonizing isolates. The results from the present study underscored the importance of the oral cavity as reservoir of staphylococci with diverse genetic traits related to human disease.

## 4. Materials and Methods

### 4.1. Study Design

This was an observational, cross-sectional study conducted in dental hospitals affiliated with Health Sciences University of Hokkaido, Ishikari-Tobetsu town, in Hokkaido, which is the northern main island of Japan. Two samples (saliva and skin swab from hand) taken from individual participants (dental patients or staff) were cultured for isolation of staphylococcus. The grown bacterial colonies were analyzed genetically for species, genotypes, virulence factors and drug resistance genes, as well as antimicrobial susceptibility testing. Informed consent was obtained from all the participants as shown below, and the confidentiality of participants’ information was ensured throughout the study, as approved by the institutional review board.

### 4.2. Study Subjects and Isolation of Staphylococcus

During the period between December 2019 and February 2021, oral (saliva) and hand (skin swab) samples were collected from dental patients and staff in two dental treatment facilities who agreed to participate to this study. Saliva specimens were collected from the floor of the mouth by using a sterile cotton swab. For hand swab specimens, a sterile cotton swab moistened with normal saline was rubbed on palms and fingers. Dental disease site samples were taken by using a sterile cotton swab. All the swab samples were directly plated on CHROMagar Staph aureus (Kanto Chemical Industry Co., Ltd., Tokyo, Japan) and incubated at 37 °C for 48 h aerobically. *Staphylococcus*-like colonies were subculture on blood agar plates followed by incubation at 37 °C overnight aerobically. Identification of bacterial species was performed by analysis of partial 16Sr RNA gene sequencing of PCR products with primers 16Sr-1: GATGAACGCTGGCGGCGTGCCT and 16Sr-2: TGTTACGACTTCACCCCAATC designed in this study. Individual isolates were stored in cryovials (Microbank, Pro-Lab Diagnostics, Richmond Hill, ON, Canada) at −80 °C and recovered when they were analyzed.

DNA samples were extracted from cultured bacterial cells by the use of achromopeptidase (FUJIFILM Wako Pure Chemical Corp., Osaka, Japan) as described previously [65]. The PCR mixture contained 200 μM dNTP, 0.5 μM each primer, 1.25 U Ex Taq DNA polymerase (Takara Bio Inc., Shiga, Japan) and its buffer with Mg^2+^ (final conc. 2 mM), extracted bacterial DNA (approximately 2–3 ng), and sterile distilled water to a final volume of 25 μL. A PCR was performed on a thermal cycler (Gene Atlas, ASTEC, Fukuoka, Japan) with the following conditions: preheating at 94 ℃ for 2 min, 30 cycles of denaturation at 94 ℃ for 15 s, annealing at 55 ℃ for 15 s and extension at 72 ℃ for 15 s, and a final extension at 72 ℃ for 3 min. PCR amplicons were analyzed for their size using electrophoresis on a 2% agarose gel. Nucleotide sequence was determined using Sanger sequencing with the PCR products using the BigDye Terminator v3.1 Cycle Sequencing kit (Applied Biosystems, Foster City, CA, USA) on an automated DNA sequencer (ABI PRISM 3100, Applied Biosystems, Foster City, CA, USA).

### 4.3. Antimicrobial Susceptibility Testing

For all the isolates, minimal inhibitory concentrations (MICs) within limited ranges were measured using a broth microdilution test using Dry Plate Eiken DP32 (Eiken, Tokyo, Japan) for the following 18 antimicrobials: oxacillin (OXA), ampicillin (AMP), cefazolin (CFZ), cefmetazole (CMZ), flomoxef (FMX), imipenem (IPM), gentamicin (GEN), arbekacin (ABK), erythromycin (ERY), clindamycin (CLI), vancomycin (VAN), teicoplanin (TEC), linezolid (LZD), minocycline (MIN), fosfomycin (FOF), levofloxacin (LVX), cefoxitin (FOX) and trimethoprim/sulfamethoxazole (SXT). MICs of GEN, ciprofloxacin (CIP), tetracycline (TET), doxycycline (DOX) and lincomycin (LIN) were measured manually using a broth microdilution test for selected isolates.

Resistance was judged according to the break points mentioned in the Clinical Laboratory Standards Institute guidelines for most of the antimicrobials tested (CLSI). For antimicrobial drugs, whose breakpoints are not defined by CLSI guidelines, we employed the European Committee on Antimicrobial Susceptibility Testing (EUCAST) breakpoint for FOF (32 μg/mL, *Staphylococcus* spp.), and a unique breakpoint for ABK (4 μg/mL, which is higher than the 2 μg/mL defined by the Japanese Society of Chemotherapy for a respiratory infection), and a breakpoint of FMX (16 μg/mL) defined by the Japanese Society of Chemotherapy for a urinary tract infection.

### 4.4. Genetic Typing

For all the isolates, the presence of *nuc*, *mecA*, PVL genes and ACME-associated *arcA* was confirmed by a multiplex PCR assay as described by Zhang et al. [66]. In addition, to discriminate species of non-*S. aureus* complex (*S. argenteus*, *S. schweitzeri*) from *S. aureus*, a PCR targeting the non-ribosomal peptide synthetase (NRPS) gene with the primers nrps-F and nrps-R was performed as described previously [67]. For all the methicillin resistant (*mecA*-positive) isolates, SCC*mec* type and subtype of SCC*mec*-IV were determined using a multiplex PCR using previously published primers and conditions [68,69]. Long-range-PCR (LR-PCR), as described previously, was applied for all the ACME *arcA*-positive strains to assign ACME type I, II, II’ [61]. Genotype of staphylocoagulase gene (*coa*) of *S. aureus* was determined by partial *coa* sequences (D1, D2 and the central regions) and analyzed for their highly similar *coa* sequence by a BLAST search (http://blast.ncbi.nlm.nih.gov/Blast.cgi, accessed on 30 April 2021) to assign *coa*-type. Accessory gene regulator (*agr*) group was assigned by the PCR with specific primers, as previously described [70]. Sequence type (ST) was determined according to the scheme of multilocus sequencing typing (MLST) [71] and sequence of protein A gene X-region (*spa* type) was determined using a PCR and direct sequencing [72], using Ridom SpaServer (http://spa.ridom.de/index.shtml, accessed on 30 April 2021) for some selected *S.aureus* isolates. PVL-encoding phages (Φ108, ΦPVL, ΦSa2958, ΦSa2MW, ΦSLT, ΦTCH60, ΦSa2usa and ΦSa119) for the PVL-positive isolates were determined using a multiplex or uniplex PCR as described previously [73].

### 4.5. Detection of Virulence Factors and Drug Resistance Genes

The presence of 28 staphylococcal enterotoxin (SE) (-like) genes (*sea-see*, *seg-selu*, selx, *sey*, *selw*, *selz*, *sel26* and *sel27*), the TSST-1 gene (*tst-1*) and exfoliative toxin genes (*eta*, *etb* and *etd*), leukocidins (*lukDE* and *lukM*), haemolysins (*hla*, *hlb*, *hld* and *hlg*), adhesin genes (*eno*, *cna*, *sdrC*, *sdrD*, *sdrE*, *fib*, *clfA*, *clfB*, *fnbA*, *fnbB*, *icaA*, *icaD*, *edinA*, *edinB*, *bap*, *spj*), modulators of host defense (*sak*, *chp* and *scn*) and ACME-I component *speG* was analyzed using multiplex or uniplex PCRs as described previously [43,74]. Genes conferring resistance to penicillin (*blaZ*), macrolides-lincosamides-streptogramins (*ermA*, *ermB*, *ermC*, *msrA*, *lnuA*, *lnuB*), aminoglycosides (*aac(6′)-Im*, *aac(6′)-Ie-aph(2”)-Ia*, *ant(3”)-Ia*, *ant(4′)-Ia*, *ant(6)-Ia*, *ant(9)-Ia*, *ant(9)-Ib*, *aph(2”)-Ib*, *aph(2”)-Ic*, *aph(2”)-Id* and *aph(3′)-IIIa*), linezolid (*optrA*) and chloramphenicol (*cfr*) were detected using a uniplex or multiplex PCR using the primers previously reported [43,75]. A PCR was performed as described above (4.2), while the annealing temperature of a PCR was different depending on the target gene.

### 4.6. Statistical Analysis

Statistical analyses were performed using IBM SPSS Statistics ver.26. The chi-square test was used to analyze the differences in the prevalence of bacterial attribute information among identified STs. A *p*-value < 0.05 was considered statistically significant.

## Figures and Tables

**Table 1:** *S. aureus*, *S. argenteus* and CoNS isolates from study subjects (*n* = 133).

Study Subjects	Number of *S. aureus*/*S. argenteus*-Positive Subjects in Any Site (%)	Site	Number of Isolates [*mecA*-Positive]		
			*S. aureus*	*S. argenteus*	CoNS
staffs (*n* = 42)	16 (38.1)	oral cavity (saliva)	14 [1]		6
		hand (skin)	10		37
Patients with mild dental disease (*n* = 74)	34 (45.9)	oral cavity (saliva)	30 [2]	3	5 [1]
		hand (skin)	13	1	79 [7]
Patients with severe dental disease *^1^ (*n* = 17)	9 (52.9)	oral cavity (saliva)	7		4 [1]
		dental disease site	4		6
		hand (skin)	5		25 [2]
total (*n* = 133)	59 (44.4)		83 [3]	4 [0]	162 [11]

*^1^ Patients with dental diseases included subjects with periodontitis, implantitis, deep dental caries, abscess, fistula.

**Table 2:** Prevalence of *coa* genotype, ST, drug resistance genes and antimicrobial resistance profiles of *S. aureus* (*n* = 83) and *S. argenteus* (*n* = 4) isolates.

*S. aureus*/ *S. argenteus*	*Coa* Genotype	No. of Isolates in *Coa*-Type (%)	ST (CC)	No. of Isolates in ST	SCC*mec* type [MRSA]	Antimicrobial Resistance Profile *^2,3^	Drug Resistance Genes *^4^
*S. aureus* (*n* = 83)	IIa		ST5 (CC5)	1		All susceptible	
		3 (3.6)	ST26	1		AMP	*blaZ*
			ST1607 (CC97)	1		AMP	*blaZ*
	IIIa	11 (13.3)	ST8 (CC8)	10	SCC*mec* IVl (1 isolate)	OXA (10%), FOX (10%), AMP (60%), ERY (20%) CLI-i (20%)	*blaZ* (60%), *erm(C)* (10%), *erm(A)* (10%)
			ST6562 *^1^ (CC8)	1	SCC*mec* IVa	OXA, FOX, AMP, ERY, LVX	*blaZ*, *aph(3’)-IIIa*, *msrA*
	IVa	8 (9.6)	ST30 (CC30)	8		AMP, ERY, CLI-i, GEN (12.5%)	*blaZ*, *erm(A)*, *aac(6’)-Ie-aph(2’’)-Ia* (12.5%)
	Va	6 (7.2)	ST121 (CC121)	6		AMP (66.7%), ERY (33.3%), CLI-i (33.3%), GEN (66.7%), LVX (16.7%)	*blaZ* (66.7%), *erm(C)* (33.3%), *aac(6’)-Ie-aph(2’’)-Ia* (16.7%)
	Vb	8 (9.6)	ST188 (CC1)	8		LVX (25%)	
	VIc	10 (12.0)	ST97 (CC97)	10		AMP (20%)	*blaZ* (20%)
	VIIa	11 (13.3)	ST12 (CC12)	8		All susceptible	
			ST81 (CC1)	2		AMP, ERY, CLI-i	*blaZ*, *erm(A)*
			ST4775 (CC1)	1	SCC*mec* IVa	OXA, FOX, AMP, ERY, CLI-i	*blaZ*, *erm(A)*
	VIIb	9 (10.8)	ST45 (CC45)	5		AMP (20%)	*blaZ* (20%)
			ST508 (CC45)	2		All susceptible	
			ST291 (CC398)	1		All susceptible	
			ST398 (CC398)	1		ERY, CLI-i	*erm(C)*
	VIIIa	4 (4.8)	ST20 (CC20)	4		AMP	*blaZ*
	Xa	12 (14.5)	ST15 (CC15)	11		AMP (91%), GEN (36.7%)	*blaZ* (91%), *aac(6’)-Ie-aph(2’’)-Ia* (36.7%)
			ST718	1		ERY, CLI-i	*erm(A)*
	XIc	1 (1.2)	ST109 (CC1)	1		AMP, ERY, CLI-i	*blaZ*, *erm(A)*
*S. argenteus* (*n* = 4)	XId	2 (50)	ST2250	2		All susceptible	
	XV	2 (50)	ST1223	2		All susceptible	

*^1^ Novel ST detected in this study. This isolate had PVL phage ΦSa2USA and ACME-I (USA300 related clone). *^2^ Abbreviations: ABK, arbekacin; AMP, ampicillin; CFZ, cefazolin; CLI, clindamycin; CMZ, cefmetazole; ERY, erythromycin; FMX, flomoxef; FOF, fosfomycin; FOX, cefoxitin; GEN, gentamicin; IPM, imipenem; LVX, levofloxacin; MIN, minocycline; OXA, oxacillin; SXT, sulfamethoxazole-trimethoprim; CLI-i, inducible resistance to clindamycin (confirmed by D-zone test). *^3^ When a resistance gene is not present in all isolates of the same ST, prevalence (%) is indicated in parentheses. None of the isolates showed resistance to ABK, CFZ, CMZ, FMX, IPM, MIN, FOF, SXT, LZD (linezolid), TEC (teicoplanin) and VAN (vancomycin).*^4^ The following genes were not detected in any isolates: e*rm(B), ant(6)-Ia, aac(6’)-Im, ant(9)-Ia, ant(9)-Ib, ant(3”)-Ia, aph(2”)-Ib, aph(2”)-Ic, aph(2”)-Id, optr-A, cfr* and *fexA.*

**Table 3:** Characteristics of S. aureus / S. argenteus isolated from both oral cavity and hand of subjects (*n* = 19).

No.	Age/Sex	Isolate ID	Subject Category *^1^	Site (Sample)	*S.aureus*/*S.argenteus*	*Coa* Type	ST (CC)	Antimicrobial Resistance Profile	Drug Resistance Genes
1	26/M	A20-KT	1	saliva	*S.aureus*	VIIa	ST20 (CC20)	AMP	*blaZ*
				hand	*S.aureus*	VIIa	ST20 (CC20)	AMP	*blaZ*
2	25/M	A20-IHB	1	saliva	*S.aureus*	Vb	ST188 (CC1)	All susceptible	
				hand	*S.aureus*	Vb	ST188 (CC1)	All susceptible	
3	25/M	A20-EK	1	saliva	*S.aureus*	Va	ST121 (CC121)	AMP, GEN	*blaZ, aac(6’)-Ie-aph(2’’)-Ia*
				hand	*S.aureus*	Va	ST121 (CC121)	AMP, GEN	*blaZ, aac(6’)-Ie-aph(2’’)-Ia*
4	26/M	B20-KF	1	saliva	*S.aureus*	VIIb	ST45 (CC45)	AMP	*blaZ*
				saliva	*S.aureus*	IIIa	ST8 (CC8)	AMP	*blaZ*
				hand	*S.aureus*	XIc	ST109 (CC1)	AMP, ERY, CLI-i	*blaZ*, *erm(A)*
5	28/M	B20-HS	1	saliva	*S.aureus*	IIIa	ST8 (CC8)	All susceptible	
				hand	*S.aureus*	IIIa	ST8 (CC8)	All susceptible	
6	29/M	A21-OY	1	saliva	*S.aureus*	IIIa	ST8 (CC8)	All susceptible	
				hand	*S.aureus*	IIIa	ST8 (CC8)	All susceptible	
7	28/M	A21-OYK	1	saliva	*S.aureus*	Xa	ST15 (CC15)	All susceptible	
				hand	*S.aureus*	Xa	ST15 (CC15)	All susceptible	
8	17/M	A20-H10	2	saliva	*S.aureus*	VIIa	ST12 (CC12)	All susceptible	
				hand	*S.aureus*	VIIa	ST12 (CC12)	All susceptible	
9	9/M	A20-H16	2	saliva	*S.aureus*	VIIa	ST12 (CC12)	All susceptible	
				hand	*S.aureus*	VIc	ST97 (CC97)	All susceptible	
10	8/M	A20-H20	2	saliva	*S.aureus*	VIIa	ST12 (CC12)	All susceptible	
				hand	*S.aureus*	VIIa	ST12 (CC12)	All susceptible	
11	10F	A20-H21	2	saliva	*S.aureus*	VIIa	ST12 (CC12)	All susceptible	
				hand	*S.aureus*	VIc	ST97 (CC97)	All susceptible	
12	7/F	A20-H22	2	saliva	*S.aureus*	VIIa	ST12 (CC12)	All susceptible	
				hand	*S.aureus*	VIc	ST97 (CC97)	All susceptible	
13	8/M	A20-H24	2	saliva	*S.aureus*	VIc	ST97 (CC97)	All susceptible	
				hand	*S.aureus*	VIc	ST97 (CC97)	All susceptible	
14	9/F	A20-H40	2	saliva	*S.aureus*	VIIb	ST45 (CC45)	All susceptible	
				hand	*S.aureus*	VIIb	ST45 (CC45)	All susceptible	
15	12/F	A21-H09	2	saliva	*S.argenteus*	XId	ST2250	All susceptible	
				hand	*S.argenteus*	XId	ST2250	All susceptible	
16	10/F	B20-H05	2	saliva	*S.aureus*	Xa	ST15 (CC15)	AMP	*blaZ*
				hand	*S.aureus*	Xa	ST15 (CC15)	AMP, GEN	*blaZ, aac(6’)-Ie-aph(2’’)-Ia*
17	15/M	A20-D3	3	disease site	*S.aureus*	VIc	ST97 (CC97)	AMP	*blaZ*
				hand	*S.aureus*	VIc	ST97 (CC97)	AMP	*blaZ*
18	10/F	A20-D10	3	saliva	*S.aureus*	VIIa	ST81 (CC1)	AMP, ERY, CLI-i	*blaZ*, *erm(A)*
				hand	*S.aureus*	VIIb	ST508 (CC45)	All susceptible	
19	58/M	A21-D04	3	saliva	*S.aureus*	VIc	ST97 (CC97)	All susceptible	
				hand	*S.aureus*	VIc	ST97 (CC97)	All susceptible	

*^1^ 1, hospital staff (*n* = 7); 2, patient with mild dental disease (*n* = 9); 3, patient with severe dental disease (*n* = 3).

**Table 4:** Species of CoNS isolated from subjects.

CoNS Species	No. of Isolates [*mecA*-Positive]			
	Oral Cavity	Hand	Dental Disease Site	Total (*n* = 162) (%)
*S. warneri*	6	51 [1]	1	58 (35.8) [1]
*S. capitis*	2	46	4	52 (32.1)
*S. saprophyticus*	2 [1]	10 [4]	0	12 (7.4) [5]
*S. epidermidis*	4 [1]	4	1	9 (5.6) [1]
*S. caprae*	0	8	0	8 (5.0)
*S. haemolyticus*	0	7 [3]	0	7 (4.3) [3]
*S. cohnii*	0	5	0	5 (3.1)
*S. lugdunensis*	1	2 [1]	0	3 (1.9) [1]
*S. pasteuri*	0	2	0	2 (1.2)
*S. xylosus*	0	2	0	2 (1.2)
*S. auricularis*	0	1	0	1 (0.6)
*S. condimenti*	0	1	0	1 (0.6)
*S. hominis*	0	1	0	1 (0.6)
*S. petrasii*	0	1	0	1 (0.6)
Total	15 [2]	141 [9]	6	162 [11]

## Supplementary Materials

The following are available online at https://www.mdpi.com/article/10.3390/antibiotics10111316/s1, Table S1: Subjects of this study (*n* = 133), Table S2: Antimicrobial resistance profile of MRSA/MSSA/*S. argenteus* isolates (*n* = 87), Table S3: Genotype, drug resistance profile/gene and virulence factor in *S. aureus* (MRSA/MSSA) and *S. argenteus* isolates (*n* = 42), Table S4: Identification of SCC*mec* and ACME among staphylococcal isolates, Table S5: Antimicrobial resistance profile of CoNS isolates, Table S6: Drug resistance profile/gene in MR-CoNS (*n* = 11).
