# Supplementary material for: Antimicrobial Resistance and Molecular Epidemiological Characteristics of Methicillin-Resistant and Susceptible Staphylococcal Isolates from Oral Cavity of Dental Patients and Staff in Northern Japan
Source: Antibiotics (Basel). 2021 Oct 29;10(11):1316. doi: 10.3390/antibiotics10111316 (PMC8615198; doi:10.3390/antibiotics10111316)
Supplement: Supplementary file 1 [file antibiotics-10-01316-s001.zip › antibiotics-1423683-supplementary.pdf]

**Table S1 Subjects of this study (n=133)**

| Categories of study subjects | | Male | Female | Total |
|---|---|---|---|---|
| staff (n=42) | Dentist | 21 | 14 | 35 |
| | Dental hygienist | 1 | 6 | 7 |
| Patients without dental disease (n=74) | child (0-18 y) | 28 | 24 | 52 |
| | adult (19-84 y) | 7 | 15 | 22 |
| Patients with dental disease (n=17) | child (0-18 y) | 2 | 1 | 3 |
| | adult (19-93 y) | 7 | 7 | 14 |
| Total | | 66 | 67 | 133 |

**Table S2 Antimicrobial resistance profile of MRSA/MSSA/*S. argenteus* isolates (n=87)**

| *coa* genotype-ST (CC)/SCC*mec* | No. of isolates | No. of isolates showing resistance to antimicrobials (%) | | | | | | | |
|---|---|---|---|---|---|---|---|---|---|
| | | OXA | FOX | AMP | ERY | CLI-i | CLI-c | GEN | LVX |
| **MRSA (n=3)** | | | | | | | | | |
| *coa*-IIIa-ST8 (CC8)/SCC*mec* IVl | 1 | 1 | 1 | 1 | 1 | 1 | 0 | 1 | 0 |
| *coa*-IIIa-ST6562 (CC8)/SCC*mec* IVa | 1 | 1 | 1 | 1 | 1 | 0 | 0 | 0 | 1 |
| *coa*-VIIa-ST4775 (CC1)/SCC*mec* IVa | 1 | 1 | 1 | 1 | 1 | 0 | 0 | 0 | 0 |
| **MSSA (n=80)** | | | | | | | | | |
| *coa*-IIa-ST5 | 1 | 0 | 0 | 0 | 0 | 0 | 0 | 0 | 0 |
| *coa*-IIa-ST26 | 1 | 0 | 0 | 1 | 0 | 0 | 0 | 0 | 0 |
| *coa*-IIa-ST1607 | 1 | 0 | 0 | 1 | 0 | 0 | 0 | 0 | 0 |
| *coa*-IIIa-ST8 | 9 | 0 | 0 | 5 | 1 | 0 | 1 | 0 | 0 |
| *coa*-IVa-ST30 | 8 | 0 | 0 | 8 | 8 | 8 | 0 | 1 | 1 |
| *coa*-Va-ST121 | 6 | 0 | 0 | 4 | 2 | 2 | 0 | 4 | 1 |
| *coa*-Vb-ST188 | 8 | 0 | 0 | 0 | 0 | 0 | 0 | 0 | 2 |
| *coa*-VIc-ST97 | 10 | 0 | 0 | 2 | 0 | 0 | 0 | 0 | 0 |
| *coa*-VIIa-ST12 | 8 | 0 | 0 | 0 | 0 | 0 | 0 | 0 | 0 |
| *coa*-VIIa-ST81 (CC1) | 2 | 0 | 0 | 2 | 2 | 2 | 0 | 0 | 0 |
| *coa*-VIIb-ST45 (CC45) | 5 | 0 | 0 | 1 | 1 | 1 | 0 | 0 | 0 |
| *coa*-VIIb-ST508 (CC45) | 2 | 0 | 0 | 0 | 0 | 0 | 0 | 0 | 0 |
| *coa*-VIIb-ST291 | 1 | 0 | 0 | 0 | 0 | 0 | 0 | 0 | 0 |
| *coa*-VIIb-ST398 | 1 | 0 | 0 | 0 | 1 | 1 | 0 | 0 | 0 |
| *coa*-VIIIa-ST20 | 4 | 0 | 0 | 4 | 0 | 0 | 0 | 0 | 0 |
| *coa*-Xa-ST15 | 11 | 0 | 0 | 6 | 0 | 0 | 0 | 3 | 0 |
| *coa*-Xa-ST718 | 1 | 0 | 0 | 0 | 1 | 1 | 0 | 0 | 0 |
| *coa*-XIc-ST109 | 1 | 0 | 0 | 1 | 1 | 1 | 0 | 0 | 0 |
| total no. of *S. aureus* | 83 | 3 (3.6) | 3 (3.6) | 38 (45.8) | 20 (24.1) | 17 (20.5) | 1 (1.2) | 9 (10.8) | 5 (6.0) |
| ***S. argenteus* (n=4)** | | | | | | | | | |
| *coa*-XId-ST2250 | 2 | 0 | 0 | 0 | 0 | 0 | 0 | 0 | 0 |
| *coa*-XV-ST1223 | 2 | 0 | 0 | 0 | 0 | 0 | 0 | 0 | 0 |
| total no. of *S. argenteus* | 4 | 0 | 0 | 0 | 0 | 0 | 0 | 0 | 0 |

None of the isolates showed resistance to ABK, CFZ, CMZ, FMX, IPM, LZD, MIN, FOF, SXT, TEC and VAN.

Abbreviations: ABK, Arbekacin; AMP, Ampicillin; CFZ, Cefazolin; CLI, Clindamycin; CMZ, Cefmetazole; ERY, Erythromycin; FMX, Flomoxef: FOF, Fosfomycin; FOX, Cefoxitin; GEN, Gentamycin; IPM, Imipenem; LVX, Levofloxacin; LZD, Linezolid; MIN, Minocycline; OXA, Oxacillin; SXT, Sulfamethoxazole-Trimethoprim; TEC, Teicoplanin; VAN, Vancomycin.

# Table S3 Genotype, drug resistance profile/gene, and virulence factor in *S. aureus* (MRSA/MSSA) and *S. argenteus* isolates (n=42)

| MRSA / MSSA / *S. argenteus* | genotype | | | | Subject | | | Antimicrobial resistance | | Virulence factors | |
|---|---|---|---|---|---|---|---|---|---|---|---|
| | SCC*mec* | *coa* | ST (CC) | *agr* [*1] | ID | Specimen / category [*2] | Age / sex | Profile [*3] | Resistance genes [*4] | Leukocidins, haemolysins, enterotoxins, TSST-1 [*5] | Adhesins, Modulators of host defense, ACME [*5] |
| MRSA | IVl | IIIa | ST8 (CC8) | I | B20-H09 | saliva / 2 | 13/M | OXA, FOX, AMP, ERY, GEN | *blaZ, ermC, aac(6')-Ie-aph(2'')-Ia, ant(4')* | *lukDE, hlb, hlg, sec, sep, tst-1* | *eno, ebpS, sdrC, sdrD,sak, scn, spj* |
| | IVa | IIIa | ST6562*(CC8) | I | A20-H47 | saliva / 2 | 66/F | OXA, FOX, AMP, ERY, LVX | *blaZ, aph(3')-IIIa, msr(A)* | *lukS-PV-lukF-PV* (ΦSa2usa), *lukDE, hlb, hlg* | *fib, eno, cna, ebpS, sak, chp, speG*, ACME-I |
| | IVa | VIIa | ST4775 (CC1) | III | A21-MT | saliva / 1 | 38/M | OXA, AMP, FOX, ERY,CLI-i | *blaZ, erm(A)* | *lukDE, hlb* | *fib, ebpS, eno, sdrC, sdrD, sdrE* |
| MSSA | | IIa | ST5 | II | A20-H44 | saliva / 2 | 4/M | All susceptible | | *lukDE, hlb, hlg, seg, sei, sem, sen, seo* | *fib, ebpS, eno, sdrC, sdrE, sak, scn* |
| | | IIa | ST26 | NT | A20-D05 | saliva / 2 | 62/F | AMP | *blaZ* | *lukDE, hlg, seb, seg, sei, sem, sen, seo, etd* | *ednB, fib, ebpS, eno, sdrC, sdrD, sak, chp, scn* |
| | | IIa | ST1607 | NT | A20-H17 | saliva / 2 | 7/M | AMP | *blaZ* | *lukDE, hlb, hlg, seg, sei, sem, sen, seo, etd* | *ednB, fib, ebpS, eno, sdrC, sdrD, sdrE* |
| | | IIIa | ST8 | I | A20-H31 | saliva / 2 | 24/F | AMP | *blaZ* | *lukDE, hlb, hlg, sea* | *fib, ebpS, eno, sdrC, sdrD, sdrE, sak, scn* |
| | | IIIa | ST8 | I | A19-FY | hand / 1 | 32/M | AMP | *blaZ* | *lukDE, hlb, hlg, sea* | *fib, ebpS, eno, sdrC, sdrD, sdrE, sak, scn* |
| | | IIIa | ST109 | II | B20-KF | hand / 1 | 26/M | AMP, ERY | *blaZ, erm(A)* | *hlb, hlg, seg, sei, sem, sen, seo* | *fib, ebpS, eno, sdrC, sdrD, sak, chp, scn* |
| | | IVa | ST30 | III | B20-H08 | saliva / 2 | 16/M | AMP, ERY, GEN, LVX | *blaZ, erm(A), aac(6')-Ie-aph(2'')-Ia* | *hlb, hlg, seg, sei, sem, sen, seo, seu, tst-1* | *ebpS, eno, cna, sdrC, sak, chp, scn* |
| | | IVa | ST30 | III | A20-D01 | saliva / 3 | 25/F | AMP, ERY | *blaZ, erm(A)* | *hlb, hlg, sea, seg, sei, sem, sen, seo, seu, tst-1* | *ebpS, eno, cna, sdrC, sak, chp, scn* |
| | | IVa | ST30 | III | A20-D01 | disease site / 3 | 25/F | AMP, ERY | *blaZ, erm(A)* | *hlb, hlg, sea, seg, sei, sem, sen, seo, seu, tst-1* | *ebpS, eno, cna, sdrC, sak, chp, scn* |
| | | Va | ST121 | IV | B20-H06 | saliva / 2 | 10/M | AMP, ERY | *blaZ, erm(C)* | *lukDE, hlb, seg, sei, sem, sen, seo, seu* | *ednA, fib, ebpS-v, eno, cna, sak, scn* |
| | | Va | ST121 | IV | A20-H37 | saliva / 2 | 3/M | AMP, GEN, LVX | *blaZ, aac(6')-Ie-aph(2'')-Ia* | *lukDE, hlb, seg, sei, sem, sen, seo, seu, eta* | *fib, ebpS-v, eno, cna, sak, scn* |
| | | Va | ST121 | IV | A20-EK | saliva / 1 | 25/M | AMP, GEN | *blaZ, aac(6')-Ie-aph(2'')-Ia* | *lukDE, hlb, seg, sei, sem, sen, seo, seu, eta* | *fib, ebpS-v, eno, sak, scn* |
| | | Vb | ST188 | I | A20-FA | saliva / 1 | 45/M | All susceptible | | *lukDE, hlb* | *fib, ebpS, eno, cna, sdrC, sdrE, sak, chp, scn* |
| | | Vb | ST188 | I | A20-YT | saliva / 1 | 34/M | LVX | | *lukDE, hlb* | *fib, ebpS, eno, cna, sdrC, sdrE, sak, chp, scn* |
| | | Vb | ST188 | I | A20-YK | saliva / 1 | 37/M | All susceptible | | *lukDE, hlb* | *fib, ebpS, eno, sdrC, sdrD, sdrE, sak, scn* |
| | | VIc | ST97 | I | A20-D03 | disease site / 3 | 15/M | AMP | *blaZ* | *lukDE, hlb* | *fib, ebpS, eno, sdrC, sdrD, sdrE, sak, scn* |
| | | VIc | ST97 | I | A20-D03 | hand / 3 | 15/M | AMP | *blaZ* | *lukDE, hlb* | *fib, ebpS, eno, sdrC, sdrD, sdrE, sak, scn* |
| | | VIc | ST97 | I | A20-H24 | saliva / 2 | 8/M | All susceptible | | *lukDE, hlb* | *fib, ebpS, eno, sdrC, sdrD, sdrE, sak, scn* |
| | | VIc | ST97 | I | A20-H24 | hand / 2 | 8/M | All susceptible | | *lukDE, hlb* | *fib, ebpS, eno, sdrC, sdrD, sdrE, sak, scn* |
| | | VIc | ST97 | I | A20-H22 | hand / 2 | 7/F | All susceptible | | *lukDE, hlb* | *fib, ebpS, eno, sdrC, sdrD, sdrE, sak, scn* |
| | | VIc | ST97 | I | A20-H16 | hand / 2 | 9/M | All susceptible | | *lukDE, hlb* | *fib, ebpS, eno, sdrC, sdrD, sdrE, sak, scn* |
| | | VIIa | ST81 | III | A20-H38 | saliva / 2 | 12/F | AMP, ERY | *blaZ, erm(A)* | *lukDE, seb* | *fib, eno, cna, sdrC, sdrD, sdrE, sak, chp, scn* |
| | | VIIa | ST12 | II | A20-H22 | saliva / 2 | 10/F | All susceptible | | *lukDE, hlb, seb* | *fib, ebpS, eno, cna, sdrC, sak, chp, scn* |
| | | VIIa | ST12 | II | A20-H16 | saliva / 2 | 9/M | All susceptible | | *lukDE, hlb, seb* | *fib, ebpS, eno, cna, sdrC, sak, scn* |
| | | VIIb | ST45 | I | B20-KF | saliva / 1 | 26/M | AMP | *blaZ* | *lukDE, hlb, hlg, seg, sei, sem, sen, seo* | *fib, ebpS, eno, cna, sak, chp, scn* |
| | | VIIb | ST508 | I | A20-D10 | hand / 3 | 10/F | All susceptible | | *lukDE, hlb, hlg, seg, sei, sem, sen, seo* | *fib, ebpS, eno, cna, sak, chp, scn* |
| | | VIIb | ST291 (ST398 DLV) | I | A20-YM | saliva / 1 | 28/M | All susceptible | | *lukDE, hlb, hlg, etd* | *ednB, ebpS, sdrC, sak, chp* |
| | | VIIb | ST398 | I | A21-H02 | hand / 2 | 61/M | ERY, CLI-i | *erm(C)* | *hlb, hlg* | *eno, ebpS, sdrC, chp* |
| | | VIIIa | ST20 | I | A20-H49 | saliva / 2 | 8/M | AMP | *blaZ* | *lukDE, hlb, seg, sei, sem, sen, seo* | *fib, ebpS, eno, sdrC, sdrD, sdrE, sak, chp, scn* |
| | | VIIIa | ST20 | I | KT | saliva / 1 | 26/M | AMP | *blaZ* | *lukDE, hlb, seg, sei, sem, sen, seo* | *fib, ebpS, eno, sdrC, sdrD, sdrE, sak, chp, scn* |
| | | Xa | ST15 | II | B20-H03 | saliva / 2 | 39/F | AMP | *blaZ* | *lukDE, hlb* | *fib, ebpS, eno, sdrC, sdrD, sdrE, chp, scn* |
| | | Xa | ST15 | II | B20-H05 | hand / 2 | 10/F | AMP, GEN | *blaZ, aac(6')-Ie-aph(2'')-Ia* | *lukDE, hlb* | *fib, ebpS, eno, sdrC, sdrD, sdrE, chp, scn* |
| | | Xa | ST15 | II | B20-H05 | saliva / 2 | 10/F | AMP | *blaZ* | *lukDE, hlb* | *fib, ebpS, eno, sdrC, sdrD, sdrE, chp, scn* |
| | | Xa | ST15 | II | A20-D07 | saliva / 3 | 71/F | AMP, GEN | *blaZ, aac(6')-Ie-aph(2'')-Ia* | *lukDE, hlb* | *fib, ebpS, eno, sdrC, sdrD, sdrE, chp, scn* |
| | | Xa | ST718 | II | A20-H39 | saliva / 2 | 8/M | ERY | *erm(A)* | *lukDE, hlb* | *fib, ebpS, eno, cna, sdrC, sdrD, sdrE, sak, chp, scn* |
| *S. argenteus* | | XV | ST1223 | NT | A20-H51 | saliva / 2 | 8/F | All susceptible | | *hlb, hlg, seg, sei, sem, sen, seo, seu* | *ebpS, sdrC, sdrD, sdrE* |
| | | XV | ST1223 | NT | A20-H52 | saliva / 2 | 10/F | All susceptible | | *hlb, hlg, seg, sei, sem, sen, seo, seu* | *ebpS, sdrC, sdrD, sdrE* |
| | | XId | ST2250 | NT | A21-H09 | saliva / 2 | 12/F | All susceptible | | *sey* | *eno, sdrD, sdrE* |
| | | XId | ST2250 | NT | A21-H09 | hand / 2 | 12/F | All susceptible | | *sey* | *eno, sdrD, sdrE* |

[*1] NT, nontypeable

[*2] 1, hosptial staff (n=10); 2, patients with mild dental disease (n=26); 3, pateints with severe dental disease (n=6)

[*3] Abbreviations: ABK, arbekacin; AMP, ampicillin; CFZ, cefazolin; CLI, clindamycin; CMZ, cefmetazole; ERY, erythromycin; FMX, flomoxef; FOF, fosfomycin; FOX, cefoxitin; GEN, gentamicin; IPM, imipenem; LVX, levofloxacin; MIN, minocycline; OXA, oxacillin; SXT, sulfamethoxazole-trimethoprim; CLI-i, inducible resistance to clindamycin (confirmed by D-zone test). None of the isolates showed resistance to ABK, CFZ, CMZ, FMX, IPM, LZD, MIN, FOF, SXT, TEC and VAN.

[*4] The following genes were not detected in any isolates: *erm(B)*, *ant(6)-Ia*, *aac(6')-Im*, *ant(9)-Ia*, *ant(9)-Ib*, *ant(3")-Ia*, *aph(2")-Ib*, *aph(2")-Ic*, *aph(2")-Id*, *optr-A*, *cfr* and *fexA*.

[*5] The following genes were detected in all the strains: *hla*, *hld*, *icaA*, *icaD*, *clfA*, *clfB*. The following genes were not detected in any strain: *lukM*, *sed*, *see*, *etb*.

## Table S4 Identification of SCC*mec* and ACME among staphylococcal isolates

| SCC*mec*/ACME type | *S. aureus* (n=83) | *S. capitis* (n=52) | *S. epidermidis* (n=9) | *S. saprophyticus* (n=12) | *S. haemolyticus* (n=7) | *S. warneri* (n=58) | *S. lugdunensus* (n=3) |
|---|---|---|---|---|---|---|---|
| SCC*mec* I | 0 | 0 | 0 | 1 | 0 | 0 | 0 |
| SCC*mec* IVa | 2 | 0 | 0 | 0 | 0 | 1 | 0 |
| SCC*mec* IVl | 1 | 0 | 0 | 0 | 0 | 0 | 0 |
| SCC*mec* V | 0 | 0 | 0 | 0 | 1 | 0 | 1 |
| SCC*mec* NT* | 0 | 0 | 1 | 4 | 2 | 0 | 1 |
| total no. of isolates (%) | 3 (3.6) | 0 (0) | 1 (11.1) | 5 (41.7) | 3 (42.9) | 1 (1.7) | 2 (66.7) |
| ACME-I | 1 | 1 | 1 | 0 | 0 | 0 | 0 |
| ACME-II | 0 | 5 | 4 | 0 | 0 | 0 | 0 |
| ACME-II' | 0 | 17 | 0 | 0 | 0 | 0 | 0 |
| ACME NT* | 0 | 3 | 1 | 0 | 0 | 0 | 0 |
| total no. of isolates (%) | 1 (1.2) | 26 (50) | 6 (66.7) | 0 (0) | 0 (0) | 0 (0) | 0 (0) |

*Non-typeable

## Table S5 Antimicrobial resistance profile of CoNS isolates

| Staphylococci species | No. of isolates | *mecA*-positive | Number of isolates showing resistance to antimicrobials (%) | | | | | | | | | |
|---|---|---|---|---|---|---|---|---|---|---|---|---|
| | | | OXA | FOX | AMP | ERY | CLI-i | CLI-c | GEN | MIN | FOF | LVX |
| *S.warneri* | 58 | 1 | 1 | 1 | 12 | 17 | 4 | 1 | 8 | 1 | 30 | 0 |
| *S.capitis* | 52 | | 0 | 0 | 2 | 2 | 0 | 0 | 0 | 0 | 28 | 0 |
| *S.saprophyticus* | 12 | 5 | 5 | 5 | 5 | 6 | 1 | 0 | 0 | 0 | 8 | 1 |
| *S.caprae* | 8 | | 0 | 0 | 0 | 0 | 0 | 0 | 1 | 0 | 5 | 0 |
| *S.epidermidis* | 9 | 1 | 1 | 1 | 1 | 3 | 1 | 0 | 1 | 0 | 1 | 0 |
| *S.haemolyticus* | 7 | 3 | 2 | 3 | 3 | 3 | 2 | 2 | 0 | 0 | 3 | 3 |
| *S.cohnii* | 5 | | 0 | 0 | 2 | 3 | 0 | 1 | 0 | 0 | 0 | 0 |
| *S.lugdunensis* | 3 | 1 | 0 | 1 | 2 | 0 | 0 | 0 | 1 | 0 | 0 | 0 |
| *S.pasteuri* | 2 | | 0 | 0 | 0 | 0 | 0 | 0 | 1 | 0 | 0 | 0 |
| *S.xylosus* | 2 | | 0 | 0 | 0 | 0 | 0 | 0 | 0 | 0 | 0 | 0 |
| *S.auricularis* | 1 | | 0 | 0 | 0 | 0 | 0 | 0 | 0 | 0 | 0 | 0 |
| *S.condimenti* | 1 | | 0 | 0 | 0 | 0 | 0 | 0 | 0 | 0 | 0 | 0 |
| *S.hominis* | 1 | | 0 | 0 | 0 | 0 | 0 | 0 | 0 | 0 | 0 | 0 |
| *S.petrasii* | 1 | | 0 | 0 | 0 | 0 | 0 | 0 | 0 | 0 | 1 | 0 |
| total | 162 | 11 | 9 (5.6) | 11 (6.8) | 27 (16.7) | 34 (21) | 8 (4.9) | 4 (2.5) | 12 (7.4) | 1 (0.6) | 76 (46.9) | 4 (2.5) |

None of the isolates showed resistance to ABK, CFZ, CMZ, FMX, IPM, LZD, SXT, TEC and VAN.

Abbreviations: ABK, Arbekacin; AMP, Ampicillin; CFZ, Cefazolin; CLI, Clindamycin; CMZ, Cefmetazole; ERY, Erythromycin; FMX, Flomoxef: FOF, Fosfomycin; FOX, Cefoxitin; GEN, Gentamycin; IPM, Imipenem; LVX, Levofloxacin; LZD, Linezolid; MIN, Minocycline; OXA, Oxacillin; SXT, Sulfamethoxazole-Trimethoprim; TEC, Teicoplanin; VAN, Vancomycin.

## Table S6 Drug resistance profile/gene in MR-CoNS (n=11)

| Subject ID | Specimen / subject category[*1] | Age/sex | species | SCC*mec* Type[*2] | ACME type | Antimicrobial resistance profile[*3] | Drug resistance genes[*3] |
|---|---|---|---|---|---|---|---|
| B20-H02-S | saliva / 2 | 7/F | *S.saprophyticus* | NT (classA *mec*) | | OXA, FOX, AMP, ERY, FOF | *blaZ*, *msrA* |
| A20-H44-h1 | hand / 2 | 4/M | *S.saprophyticus* | NT (classA *mec*) | | FOX, OXA, AMP, ERY, FOF | *blaZ*, *msrA* |
| B20-H02-h2 | hand / 2 | 7/F | *S.saprophyticus* | NT (classA *mec*) | | OXA, FOF | |
| B20-H01-h2 | hand / 2 | 34/F | *S.saprophyticus* | NT (classA *mec*, *ccrC* (+)) | | OXA, FOX, AMP, ERY, LVX | *blaZ*, *msrA* |
| A20-H36-h | hand / 2 | 6/F | *S.saprophyticus* | SCC*mec* I | | FOX, OXA, AMP, ERY, FOF | *blaZ*, *erm(C)* |
| B20-H01-h1 | hand / 2 | 34/F | *S.haemolyticus* | NT (classA *mec*, *ccrC* (+)) | | OXA, FOX, AMP, ERY, LVX | *blaZ*, *erm(A)* |
| A20-H35-h2 | hand / 2 | 83/F | *S.haemolyticus* | NT (classA *mec*, *ccrC* (+)) | | FOX, AMP, ERY, LVX | *blaZ*, *erm(A)* |
| A21-D01-h1 | hand / 3 | 63/M | *S.haemolyticus* | SCC*mec* V | | OXA, FOX, AMP, CLI, LVX | *blaZ* |
| A20-D04-S1 | saliva / 3 | 76/F | *S.epidermidis* | NT | ACME-I | OXA, FOX, AMP, GEN, ERY, FOF | *blaZ, erm(B)*, *aac(6')-Ie-aph(2")-Ia* |
| A20-D07-h2 | hand / 3 | 71/F | *S.lugdunensis* | SCC*mec* Va | | AMP, FOX, GEN | *blaZ, aac(6')-Ie-aph(2")-Ia* |
| A20-H23-h | hand / 2 | 5/M | *S.warneri* | SCC*mec* IV | | AMP, OXA, FOX, GEN, ERY | *blaZ, msrA*, *aac(6')-Ie-aph(2")-Ia*, *ant(9)-Ia* |

[*1] 1, hosptial staff ; 2, patient with mild dental disease ; 3, pateint with severe dental disease

[*2] NT, nontypeable

[*3] See footnote in Table S3
